# Systemic Dissemination of Tuberculosis from a Patch of Lupus Vulgaris

**Published:** 1895-10

**Authors:** 


### Editorial, Etc.

SYSTEMIC DISSEMINATION OF TUBERCULOSIS FROM A PATCH OF LUPUS VULGARIS.—Dr. Robert Peter, of Toledo, Ohio, thus writes in the Medical Review: The patient was a white man, aged fifty-eight, who had suffered from a patch of lupus valgaria, situated just below the left ear, for ten years. The patch gradually spread until it destroyed the lobule of the ear and the tissues in front of the auricle. It finally broke through the junction of the aural cartilage and penetrated the anterior temporal vein. The hemorrhage was considerable; but was finally fully controlled, and the patient continued for a year without further change. At the end of that time he had an attack which was diagnosed typhoid fever, and from which he seemed never to fully recover. His condition passed into one of peevishness, slight hectic fever, colliquative sweats, and emaciation. Then there was developed a synovites of the knee, with thickening of the condyles of the femur, and later a cold abscess appeared in the renal region, which was evacuated. Both these processes were probably tubercular. Physical examination of the chest did not reveal anything positive. The important lesson taught us by this case is that general tuberculosis can be established by dissemination from a local process of lupus vulgaris, and this lesion should always be designated as tuberculosis of the skin. The author asks the very pertinent question, Is it not probable that the lupus patch was engrafted by innoculation from a contaminated razor? The matter awakens practical interest by the relation it bears to the arguments in favor of requiring barbers to sterilize their razors and sponges.

